# Bridging-mediated compaction of mitotic chromosomes

**DOI:** 10.1080/19491034.2025.2497765

**Published:** 2025-05-09

**Authors:** Giada Forte, Lora Boteva, Nick Gilbert, Peter R. Cook, Davide Marenduzzo

**Affiliations:** a SUPA, School of Physics and Astronomy, University of Edinburgh, Edinburgh, UK; b MRC Human Genetics Unit, Institute of Genetics and Cancer, University of Edinburgh, Western General Hospital, Edinburgh, UK; c Sir William Dunn School of Pathology, University of Oxford, Oxford, UK

**Keywords:** Bridging activity, chromatin, condensin, mitotic compaction, common fragile sites

## Abstract

Within living cells, chromosome shapes undergo a striking morphological transition, from loose and uncondensed fibers during interphase to compacted and cylindrical structures during mitosis. ATP driven loop extrusion performed by a specialized protein complex, condensin, has recently emerged as a key driver of this transition. However, while this mechanism can successfully recapitulate the compaction of chromatids during the early stages of mitosis, it cannot capture structures observed after prophase. Here we hypothesize that a condensin bridging activity plays an additional important role, and review evidence – obtained largely through molecular dynamics simulations – that, in combination with loop extrusion, it can generate compact metaphase cylinders. Additionally, the resulting model qualitatively explains the unusual elastic properties of mitotic chromosomes observed in micromanipulation experiments and provides insights into the role of condensins in the formation of abnormal chromosome structures associated with common fragile sites.

## Introduction

**Figure 1.**
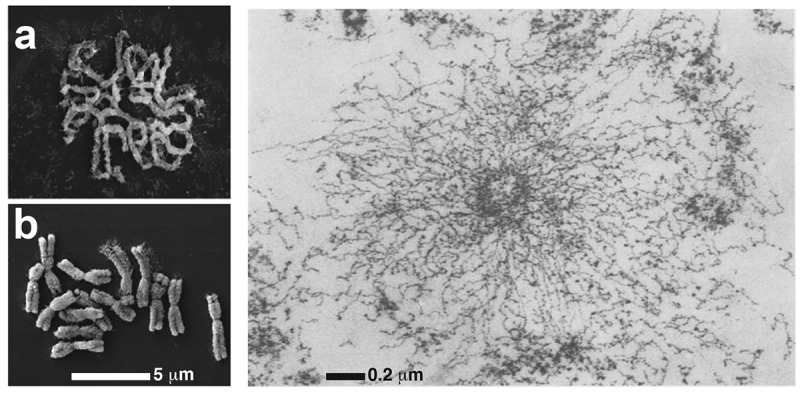
Structure of mitotic chromosomes. (a,b) Scanning electron microscopy images of mitotic rye chromosomes in prophase (panel a) and metaphase (panel b). Figure adapted from [5]. (c) Radial section of a mitotic chromosome immersed in a low salt buffer. A bottle-brush structure appears, with a central scaffold and diverging bristles. Figure adapted from ref [6].

During mitosis, transcription largely ceases, chromatin condenses [1–3], and an apparently disorganized flexible fiber compacts into a structured cylindrical chromatid [1,4] (Figure 1(a,b)). Despite substantial progress, the mechanisms driving this transition remain only partially explained ([7,8]. For example, in the late 1970s, Laemmli, one of the pioneers in this field, immersed mitotic chromosomes in a low-salt buffer, causing them to swell and reveal loops radially attached to a central scaffold [6] (Figure 1(c)). This has led to the development of many models, including those involving loops-on-a-scaffold [9–11], hierarchical structures (e.g., where the 30 nm fiber folds into a 250 nm structure that then coils into the mitotic cylinder [12,13]), networks (from experiments involving micromanipulation and small-angle X-ray scattering [14,15]), and fractals [16].

More recently, 3C and Hi-C experiments – techniques detecting interactions between chromatin loci – and polymer simulations have allowed contacts between distant parts of the fiber to be mapped [17–20]. For example, one polymer model depicts mitotic chromosomes (confined within a cylindrical simulation volume) as a sequence of consecutive loops anchored to a central scaffold [17,21,22]. As this model provides excellent agreement with experimentally determined contact probabilities [17], the loop-on-a-scaffold architecture is now largely accepted as providing a good description of the mitotic structure [18]. However, a detailed understanding of the mechanisms that lead to such a structure remains elusive.

Surprisingly, electron microscopy conducted on histone-depleted metaphase chromosomes revealed that histones are not required for mitotic chromatin condensation [23,24]. Further experimental data identified two non-histone proteins critical for mitotic folding, Sc1 and Sc2, located on the mitotic chromosome scaffold [25]. Currently, Sc1 is recognized as topoisomerase II [26,27], a protein able of resolving chromatin entanglements by transiently inducing and resealing DNA breaks [28,29]. Similarly, Sc2 was found to be SMC2, a component of condensin I and condensin II complexes [30,31]. In the absence of topoisomerase II, sister chromatids fail to separate during anaphase [32], while depleting condensins I and II induces faulty condensation, and knocking them out prevents compaction [31,33].

Although structurally similar [34,35], condensins I and II play different roles [18,36,37]. Specifically, condensin I is a cytoplasmic complex that binds immediately after the nuclear envelope breaks down and later shortens the cylindrical chromosome, whereas condensin II is a nuclear protein that binds during prophase to form the scaffold and provide axial rigidity. Micromanipulation (pulling apart ends of a mitotic chromatid) unveils a discontinuous focal pattern of condensins down the scaffold [14,38], reminiscent of the chromomeres recognized by early microscopists [39]. Remarkably, these experiments also highlight the exceptionally elastic nature of mitotic chromatids: they can be stretched up to 10 times their original length, and yet relax once pulling forces are released [11,14,38,40–42]

As both topoisomerase II and condensin complexes are molecular motors that use ATP, it is widely believed that chromosome compaction is driven by active processes [43]. Moreover, active loop extrusion by condensins in vitro [44] supports the idea that compaction requires ATP hydrolysis [18,45]. Additionally, molecular dynamics simulations demonstrate that such extrusion can fold an interphase-like fiber into a prophase-like cylinder – providing that simulations are conducted in a confining cylindrical volume [21]. Consequently, the active loop-extrusion model [46] offers a compelling mechanism to explain compaction early during mitosis; however, it fails to account for condensation occurring later during prometaphase [21] and it requires an additional mechanism to impose cylindrical confinement [17,18].

The incomplete understanding of the problem prompted us to consider alternative mechanisms for the shape changes that occur after prophase, and in metaphase. The bottlebrush topology [11,22] provides a promising initial condition for the chromatin fiber, as it recapitulates prophase structure; we note that it naturally stiffens the backbone of the chromatin fiber because adjacent loops sterically exclude each other from occupying the same volume, and the resulting entropic pressure forces loops to maximize inter-loop distance [11,22]. To explain prometaphase compaction, we hypothesized that condensins could form bridges between two different chromatin segments. Although as yet unproven in a definitive way, this bridging activity is plausible, as has already been observed for cohesins, protein complexes belonging to the same SMC family as condensins [47], and it has also been considered in other related modeling work recently [48,49]. Moreover, condensin I can fold chromosomes without loop extrusion [50], and therefore presumably must possess bridging activity. In this review, we examine recent research that has investigated the roles of looping and bridging activities in condensing mitotic chromosomes.

## Bridging-mediated compaction: a polymer model for the formation of mitotic cylinders

**Figure 2.**
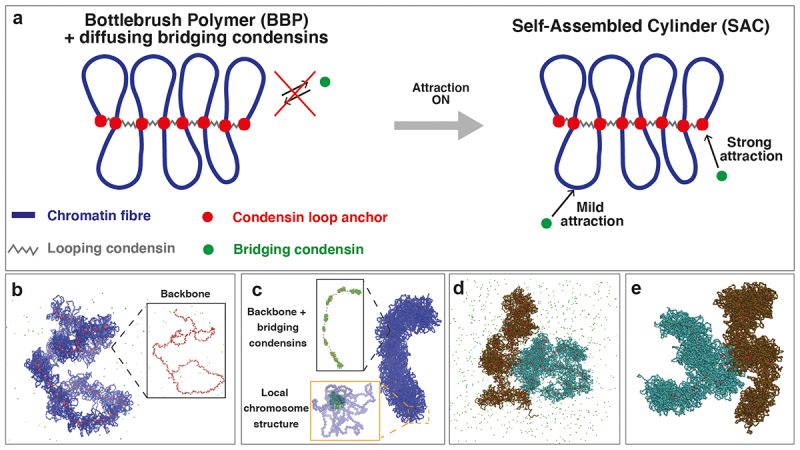
A new polymer model for mitotic chromosome folding. (a) left: Simulations start with a partially folded chromosome, represented as a bottlebrush polymer (BBP) composed by a sequence of consecutive chromatin loops (blue segments). The loop structure is due to the action of looping condensins (gray segments), each one joining the two roots of a loop, also indicated as condensin loop anchors (red beads). Bridging condensins (green beads) initially diffuse in the simulation box and experience a steric interaction with the BBP. Right: After a short time, attraction between the BBP and bridging condensins is switched on. Bridging condensins are weakly attracted to the chromatin fiber and strongly to condensin loop anchors. This attraction results in a further compaction of the mitotic chromosome, which forms a self-assembled cylinder (SAC). (b) Snapshot from a simulation representing the initial configuration (BBP + diffusing condensins). The chromosome and its backbone (inset) appear as long and flexible structures. (c) Snapshot representing a final configuration (SAC). Following the binding of bridging condensins, the mitotic chromosome is now a cylindrical and more rigid object, and concomitantly its backbone straightens and shrinks, pushing together chromatin loop anchors (bottom inset). It can be observed how bridging condensins are located along the backbone and form separated clusters (top inset). (D-E) Snapshots showing the initial and final configurations of a simulation mimicking compaction of sister chromatids, joint by a central region representing the centromere. Each chromatid is depicted as a bottlebrush polymer which compacts upon attraction with bridging condensins.

To investigate mitotic chromosome folding following prophase at kbp resolution, we describe a coarse-grained polymer model in which nucleosomal and sub-nucleosomal details are neglected [51] and whose beads are assumed to contain 2 kbp each. In our simulations, we started from a partially folded bottlebrush polymer, i.e. we assumed that the chromatin filament was arranged as a sequence of consecutive loops (Figure 2(a)) with length between 60 kbp and 120 kbp, consistent with experimental observations [17,52]. Although we did not directly simulate loop formation, we assume that these loops form during prophase via active [21,46,53] or diffusive [54,55] loop extrusion performed by condensins. We refer to the condensins involved in loop formation as looping condensins. These are modeled as springs connecting the loop bases or condensin loop anchors.

In addition to the looping activity, we introduced a new condensin bridging activity. Bridging condensins are represented as additional spheres initially diffusing into the system and experiencing steric interaction with the chromatin fiber (Figure 2a, left). Shortly after the simulation began, an attraction between the bridging condensins and chromatin was introduced. As it is reasonable to assume that looping and bridging condensins share loading sites, we assume that bridging condensins are strongly attracted to condensin loop anchors and weakly attracted to other chromatin sites (Figure 2(a), right). Because of their multivalency, bridging condensins can create contacts between two chromatin sites that lie far away along the fiber. This leads to further compaction of the chromosome which, starting from a prophase-like structure (Figure 2(b)), folds into a cylindrical object reminiscent of metaphase chromosomes, which we call self-assembled cylinder (Figure 2(c)). A similar scenario was also observed in the presence of sister chromatids: the two chromatids, initially represented as bottlebrush polymers joined together at the centromere, became compact following the binding of bridging condensins (Figure 2(d,e)).

It is also evident that the formation of a cylindrical metaphase geometry is accompanied by a change in the stiffness of the chromosome backbone, the structure formed by loop anchors. While the backbone in prophase chromosomes is long and flexible (inset in Figure 2(b)), that in metaphase chromosomes is more rigid and surrounded by clusters of bridging condensins which force chromatin loop roots to get closer to each other (insets in Figure 2(c)). Condensin clusters along the backbone of mitotic chromosomes have also been observed by microscopy, yet their origin remains unexplained [38]. In our simulations, clusters were the result of the interplay between the bridging-induced attraction mechanism and the entropic repulsion among chromatin loops. Bridging-induced attraction [56,57] arises in this case from the weak attraction between bridging condensins and chromatin. Once a condensin binds to a chromatin site, it experiences a weak interaction with other nearby sites, leading to a local increase in chromatin density. This, in turn, attracts more condensins that aggregate even without introducing an explicit attractive potential among them. However, entropic repulsion among chromatin loops prevents condensins from forming a single large cluster along the backbone [56,58]. Consequently, the condensins are forced to split, forming separate droplets. Importantly, we keep the number of bridging condensins comparable to the number of attractive loop anchors throughout. This is because too few bridging condensins would not result in mitotic condensation, whilst too many would eventually lead to the formation of a single large protein cluster which is not experimentally observed.

## Compaction and elasticity of metaphase chromosomes

**Figure 3.**
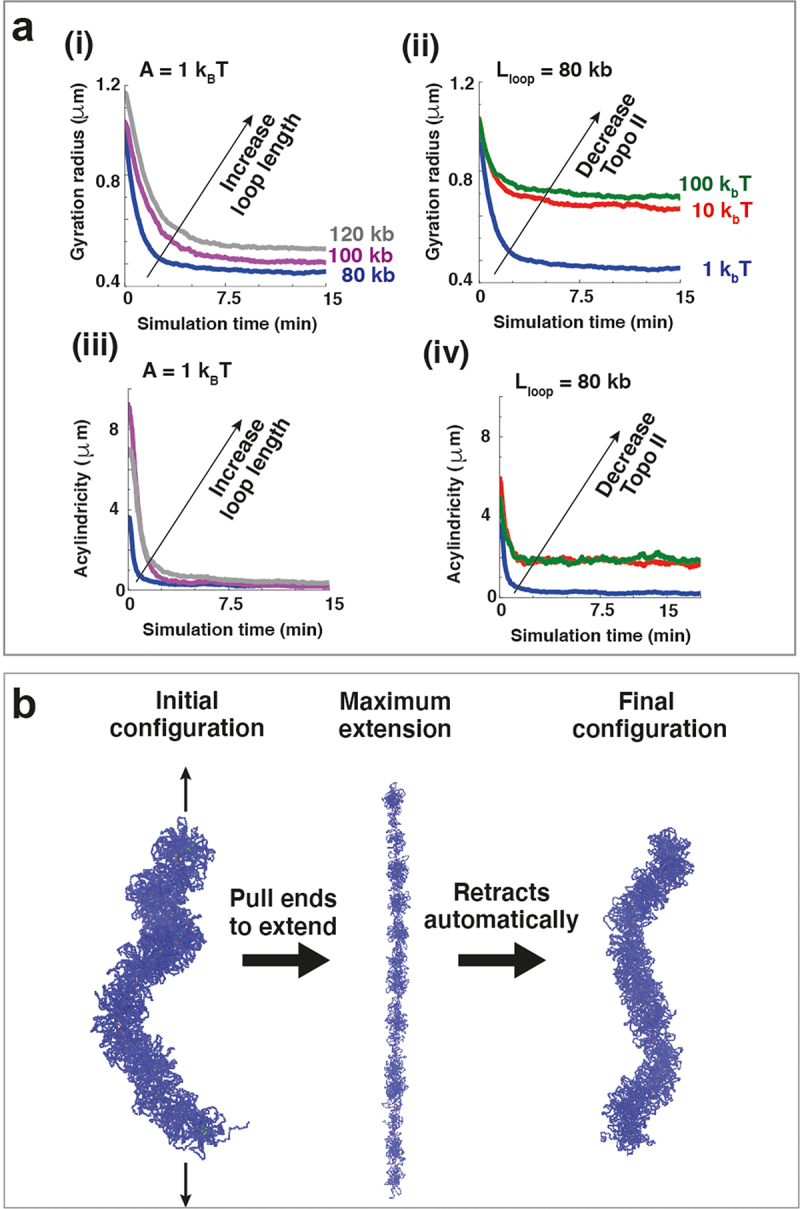
Bridging-induced compaction and mechanical properties of simulated chromosomes. (a) The bridging-induced compaction is quantified by monitoring the gyration radius (top two panels) and the acylindricity (bottom two panels) over time – i.e., while bridging condensins bind the bottlebrush polymer to form a self-assemble cylinder. Acylindricity is computed as the difference between the two smallest axes of the ellipsoid approximating the chromosome. Different curve colors refer to different values of the pair of parameters $\left(A,L_{\mathrm{loop}}\right)$, where A represents the repulsion strength among polymer beads (i.e., the level of topoisomerase II activity), and $L_{\mathrm{loop}}$ is the average length of the loops composing the chromosome. For each pair of parameters, the bottlebrush polymer compacts and assumes a more cylindrical structure as both the gyration radius and acylindricity decrease over time. We see that a better cylindrical compaction is provided by smaller loop size (panels (i) and (iii)) and high topoisomerase II activity (panels (ii) and (iv)). (b) Simulated self-assembled cylinders are elastic objects: Once equal and opposite pulling forces are applied at the chromosome extremities (left panel), the chromosome extends up to several times its original length (center panel), and yet automatically returns to an extension comparable to the initial one once the pulling forces are released (right panel).

The compaction induced by the action of bridges is quantified by monitoring the gyration radius and acylindricity as a bottlebrush polymer collapses into a self-assembled cylinder; the former parameter reflects chromatid size, and the latter reflects the deviation from a perfect cylinder. These two parameters were computed for set-ups where we varied the average length of loops composing the chromosomes, as well as topoisomerase II activity. The action of topoisomerase II was modeled by tuning the strength of the repulsion between chromatin beads. Low repulsion facilitates crossing of chromatin beads, akin to high topoisomerase II activity. In contrast, high repulsion prevents crossing among chromatin filaments, resembling low or absent topoisomerase II activity. Increasing the loop length and the concentration of topoisomerase II increased the gyration radius and decreased the acylindricity (Figure 3(a)), indicating improved cylindrical compaction. Interestingly, a significant difference in the gyration radius and acylindricity occurs when the strength of repulsion becomes comparable to thermal noise (see curves in Figure 3(a),ii, and iv referring to topoisomerase II activity of 1 k_B_T where T is the system temperature and k_B_ the Boltzmann constant). This allows chromatin beads to cross each other with relative ease, underlining the crucial role of this enzyme in the formation of compacted mitotic chromosomes.

By introducing the condensin bridging activity, we can then fold a prophase-like chromosome into a metaphase-like one, as suggested also by the contact probability curve P(s) for SAC configurations, which, for 10kbp∼ x<s∼ x<100kbp, decays as $s^{-0.5}$. The same power law is also observed in Hi-C maps of contacts within human chromosomes, although it holds for a larger range of genomic distances (100kbp∼ x<s∼ x<10Mbp) [17]. This difference can be explained by considering that we simulated chromosomes shorter than the shortest human chromosome.

Micro-manipulation experiments revealed that mitotic chromosomes are remarkably elastic; they can be stretched to several times their initial length and then relax back to the initial one [14,38,40–42,59]. The simulated self-assembled cylinders behaved similarly (Figure 3(b)). When equal and opposite forces are applied to the ends of the chromosome, its extension significantly increases; however, it returns to its original value once the pulling forces are switched off (Figure 3(b)). The condensin clusters in the backbone provided elasticity. On extension, they fragment; on relaxation, bridging-induced attraction drives reaggregation without any additional energy input, simply explaining this remarkable behavior.

## Modelling condensin knockouts and common fragile sites

By modifying the condensin bridging activity proposed here, we can also reproduce abnormal chromosome structures observed at common fragile sites or following the global or local depletion of condensin.

Experiments have shown that condensin I and II knockout result in different features: wider and shorter chromosomes for condensin I depletion, and thinner and more flexible chromosomes for condensin II depletion [60–62]. Since condensin II initiates compaction during mitosis, it is plausible to assume that its knockout could be modeled by the total removal of the looping activity, the first level of compaction in our simulations. However, eliminating chromatin loops entirely would yield a linear chromatin fiber organized around multiple bridging condensin clusters, which is not compatible with the long and flexible chromosomes observed in experiments. On the other hand, a full deletion of bridging activity would fail to replicate the characteristic features of chromosomes upon condensin I knockout, resulting instead in the bottlebrush polymer utilized as initial configuration in our simulations. Therefore, we have to hypothesize that bridging and looping activities can be performed by both condensin I and II. The balance between the two activities for each protein type might be controlled, for instance, by the overall condensin density which increases after the nuclear envelop breaks down (see Conclusions for an expanded discussion of this hypothesis).

**Figure 4.**
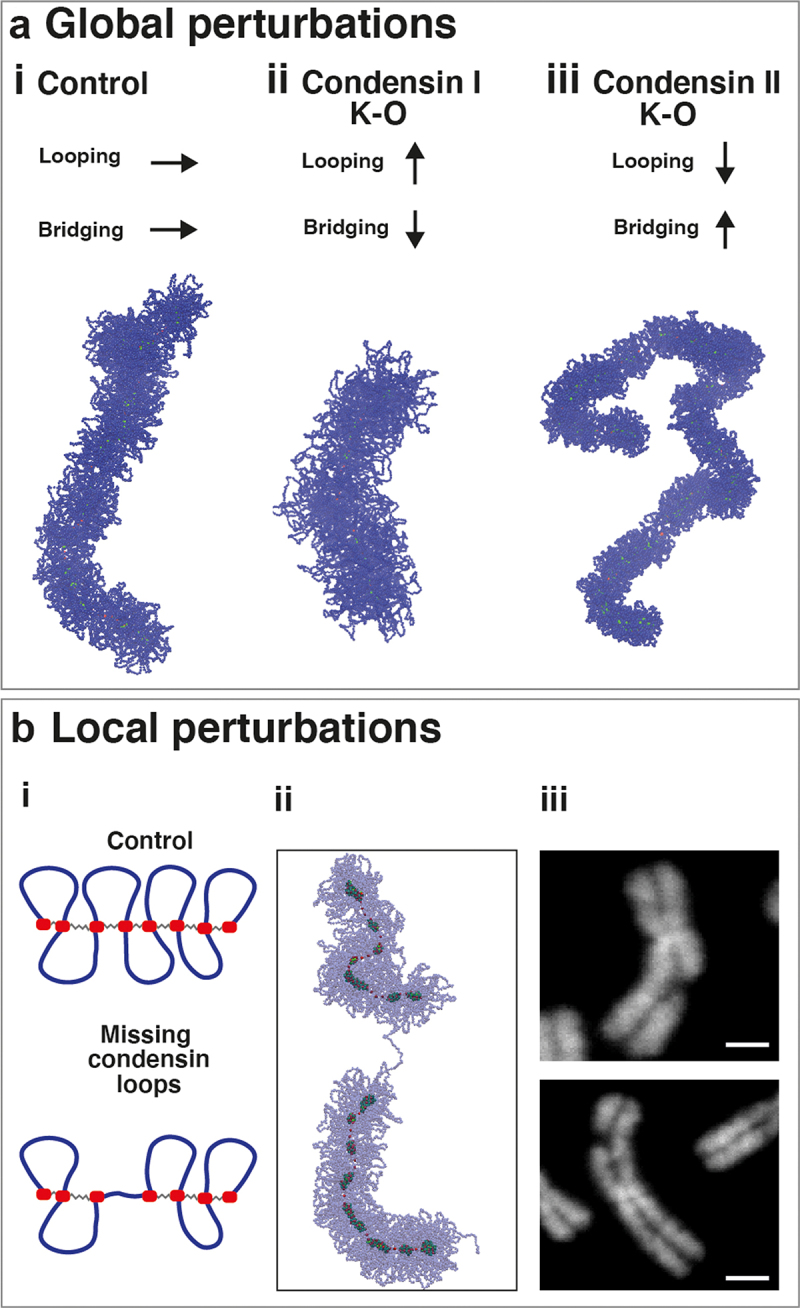
Effects of global and local perturbations of condensins. (a) Mitotic structure experimentally observed following condensin I and II knockout are reproduced by changing both the looping and bridging activity of condensins. Starting from the control self-assemble chromosome (panel (i), corresponding to wild-type conditions), condensin I knockout is simulated by increasing the looping activity and reducing the bridging activity. This results in a shorter and wider chromosome (panel (ii)). Conversely, condensin II knockout is modeled by reducing loop size and increasing the number of bridges. This leads to a longer, thinner and more flexible chromosome. (b) Local perturbations of condensins refer to faulty condensin loading in proximity of common fragile sites. An example is provided by cytological lesions, evident breakages of the mitotic chromosome (panel (iii)). This type of lesion can be replicated by changing both the looping and bridging activity, specifically by removing a few consecutive loops and the condensin binding sites corresponding to their loop anchors (panels (i) and (ii). Scale bar: 2.5μm.

Within this framework, condensin knockout can be modeled by changing the ratio between looping and bridging activity of condensins. Condensin I knockout is simulated by increasing the looping activity and reducing the bridging activity; snapshots from simulations show shorter and wider chromatids than those in the wild-type (Figure 4(a),i, and ii). Condensin II knockout is instead modeled by decreasing looping and increasing bridging; it yields longer, thinner and more flexible structures (the increased number of bridges gives shorter loops; Figure 4(a),iii). Both sets of results resemble those obtained experimentally.

Although condensin knockouts cannot be accurately replicated by completely removing one of the two condensin activities, the results of these previous simulations have revealed insights into their distinct roles. It appears that condensin II may primarily function as a loop extruder; therefore its depletion produces shorter loops and enhances the action of condensin bridges. Conversely, condensin I may predominately act as a bridge, and its deletion results in higher effective looping activity.

Interestingly, partial perturbations in the two types of activity can also successfully reproduce irregular structures observed at common fragile sites, which are often associated with the onset of cancer. An example is cytological lesions (Figure 4(b),iii) and noticeable breakages along the mitotic structure. These abnormalities can be mimicked by reducing both the looping and bridging activities of condensins, specifically by eliminating a few consecutive loops and their corresponding loop anchors (Figure 4(b),i,ii). Notably, the function of condensin bridges appears crucial for replicating the experimental structures because simply removing a few consecutive loops from a bottlebrush polymer would not result in a clear break in the mitotic cylinder.

## Conclusions

In this work we reviewed a recently proposed model to explain the folding of mitotic chromatin following prophase, which leads to an original perspective on condensin activities in mitosis. Previous *in vitro* experiments have demonstrated that condensins function as molecular motors using ATP to extrude chromatin loops [44]. Molecular dynamics simulations have also shown that such looping activity can condense an interphase-like chromosome into a prophase-like structure [21]. However, loop extrusion alone failed to yield the compact cylindrical folding observed from prometaphase onward. An alternative hypothesis discussed in [51] is that condensins can act in two different ways: as loop extruders, compacting the chromosome in a sequence of consecutive loops, or as bridges, which bind to chromatin and loop anchors via weak and strong attraction, respectively. Although the bridging activity remains speculative for the condensin complex, it has been documented in other SMC proteins, such as cohesins [47].

Within this framework, including both looping and bridging activities by condensin, a prophase chromosome modeled as a bottlebrush polymer can rearrange into a cylindrical shape, reminiscent of metaphase structures, through interactions mediated by bridging condensins. Importantly, this cylindrical rearrangement occurs spontaneously without requiring the chromosome to be placed within a cylindrical confinement, as in previous studies [17,18]. The bridging activity of condensin also naturally allows us to explain the focal clusters of condensins seen by microscopy in the axial core and the remarkable elasticity of chromatids observed during micromanipulation [38], as well as the structural variations found in the knockouts of condensins I and II [62], and the chromatid gaps observed at common fragile sites [63].

While this model provides new insights into mitotic folding, it also raises new questions.

First, what leads a bound condensin to act as a loop extruder or a bridge? We speculate that the local condensin density may determine this. Thus, lone condensins could be loop extruders; however, when concentrated, the balance may shift in favor of bridging. It is possible that condensin II binds first during mitosis to stabilize preexisting loops and/or extrude new ones (to give the initial bottlebrush polymer). Later, once the nuclear envelope breaks down, condensin I binds, which increases the density to a point that hinders extrusion and promotes bridging. In this scenario, condensins II and I would mostly function as looping and bridging condensins, respectively. Second, how do contacts observed in simulations reflect those observed by Hi-C? For example, Hi-C data point to a helical backbone [18] and polymer models have been employed to investigate this [20,64]. Although our initial bottlebrush polymers exhibited a weak helical structure (Figure 2(b), inset), this was lost as the mitotic cylinder developed (Figure 2(c), inset). Therefore, what drives the formation of helical backbones? A recent coarse-grained model proposes that the emergence of a helical structure can result from the non-equilibrium grappling motor activity of condensins, as active loop extruders [65]. Consistent with this, future studies should explore how the balance between bridging and loop extrusion activity influences the formation of the mitotic helix. Finally, in this model we did not include a non-equilibrium binding-unbinding dynamics of bridging condensins and possible condensin-condensin interactions, which may be relevant in the experiments [50]. Even if this type of interaction is not necessary to explain post-prophase folding, it will be interesting to investigate whether its presence can affect chromatin structure.

## Data Availability

Data sharing is not applicable to this article as no new data were created or analyzed in this study.
